# (1*SR*,3*RS*,3a*SR*,6a*RS*)-Methyl 5-methyl-4,6-dioxo-3-[2-(trifluoro­meth­yl)phen­yl]octa­hydro­pyrrolo­[3,4-*c*]pyrrole-1-carboxyl­ate

**DOI:** 10.1107/S1600536812051471

**Published:** 2013-01-04

**Authors:** Konstantin V. Kudryavtsev, Polina M. Ivantcova, Andrei V. Churakov

**Affiliations:** a Department of Chemistry, M.V. Lomonosov Moscow State University, Leninskie Gory 1/3, Moscow 119991, Russian Federation; b Institute of Physiologically Active Compounds, Russian Academy of Sciences, Chernogolovka 142432, Moscow Region, Russian Federation; c Institute of General and Inorganic Chemistry, Russian Academy of Sciences, Leninskii prosp. 31, Moscow 119991, Russian Federation

## Abstract

In the title compound, C_16_H_15_F_3_N_2_O_4_, the relative stereochemistry of the four stereogenic C atoms has been determined. The carb­oxy­methyl and 2-(trifluoro­meth­yl)­phenyl substituents of the pyrrolidine cycle have a *cis* mutual arrangement. The five-membered saturated aza­cycle adopts an envelope conformation with the N atom occupying the flap position. In the crystal, adjacent mol­ecules are combined in centrosymmetric dimers by two weak N—H⋯O hydrogen bonds.

## Related literature

For general background to the chemistry affording bicyclic pyrrolo­[3,4-*c*]pyrrole-based scaffolds and structural determination, see: Kudryavtsev & Irkha (2005 ▶); Kudryavtsev (2008 ▶); Kudryavtsev & Zagulyaeva (2008 ▶); Kudryavtsev *et al.* (2011 ▶).
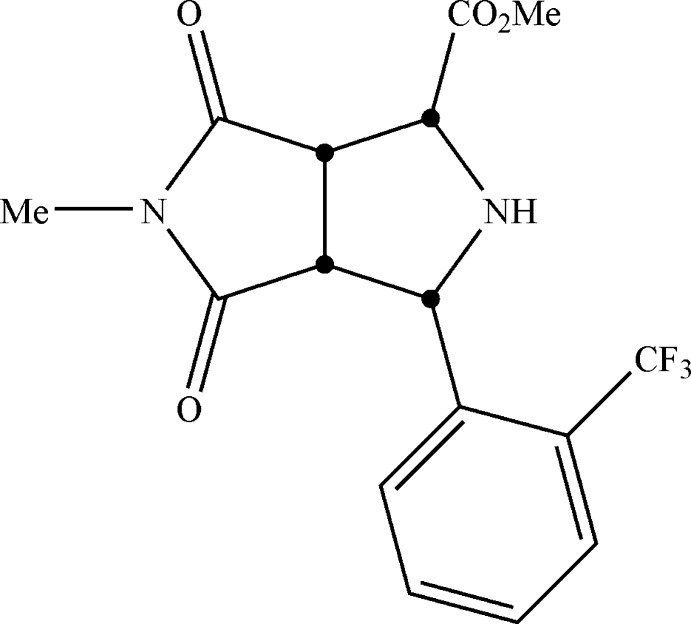


## Experimental

### 

#### Crystal data

C_16_H_15_F_3_N_2_O_4_
*M* *_r_* = 356.30
Orthorhombic, 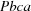

*a* = 11.6168 (4) Å
*b* = 12.7385 (5) Å
*c* = 21.2429 (8) Å
*V* = 3143.5 (2) Å^3^
*Z* = 8
Mo *K*α radiation
μ = 0.13 mm^−1^
*T* = 150 K
0.35 × 0.30 × 0.25 mm

#### Data collection

Bruker SMART APEXII diffractometer
Absorption correction: multi-scan (*SADABS*; Bruker, 2008 ▶) *T*_min_ = 0.955, *T*_max_ = 0.968
28987 measured reflections
4585 independent reflections
3698 reflections with *I* > 2σ(*I*)
*R*_int_ = 0.032

#### Refinement

*R*[*F* ^2^ > 2σ(*F* ^2^)] = 0.041
*wR*(*F* ^2^) = 0.115
*S* = 1.05
4585 reflections
286 parameters
All H-atom parameters refined
Δρ_max_ = 0.41 e Å^−3^
Δρ_min_ = −0.36 e Å^−3^

### 

Data collection: *APEX2* (Bruker, 2008 ▶); cell refinement: *SAINT* (Bruker, 2008 ▶); data reduction: *SAINT*; program(s) used to solve structure: *SHELXTL* (Sheldrick, 2008 ▶); program(s) used to refine structure: *SHELXTL*; molecular graphics: *SHELXTL*; software used to prepare material for publication: *SHELXTL*.

## Supplementary Material

Crystal structure: contains datablock(s) I, global. DOI: 10.1107/S1600536812051471/ff2094sup1.cif

Structure factors: contains datablock(s) I. DOI: 10.1107/S1600536812051471/ff2094Isup2.hkl

Supplementary material file. DOI: 10.1107/S1600536812051471/ff2094Isup3.cml

Additional supplementary materials:  crystallographic information; 3D view; checkCIF report

## Figures and Tables

**Table 1:** Hydrogen-bond geometry (Å, °)

*D*—H⋯*A*	*D*—H	H⋯*A*	*D*⋯*A*	*D*—H⋯*A*
N1—H1⋯O3^i^	0.884 (15)	2.363 (15)	3.1738 (13)	152.5 (13)

Symmetry code: (i) 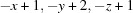.

**Fig. 1.**
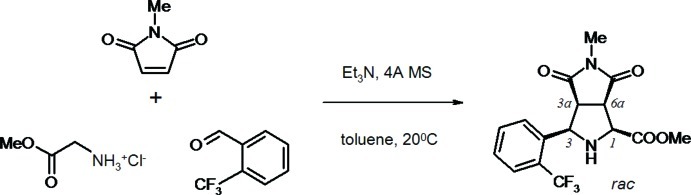
Synthetic scheme.

**Fig. 2.**
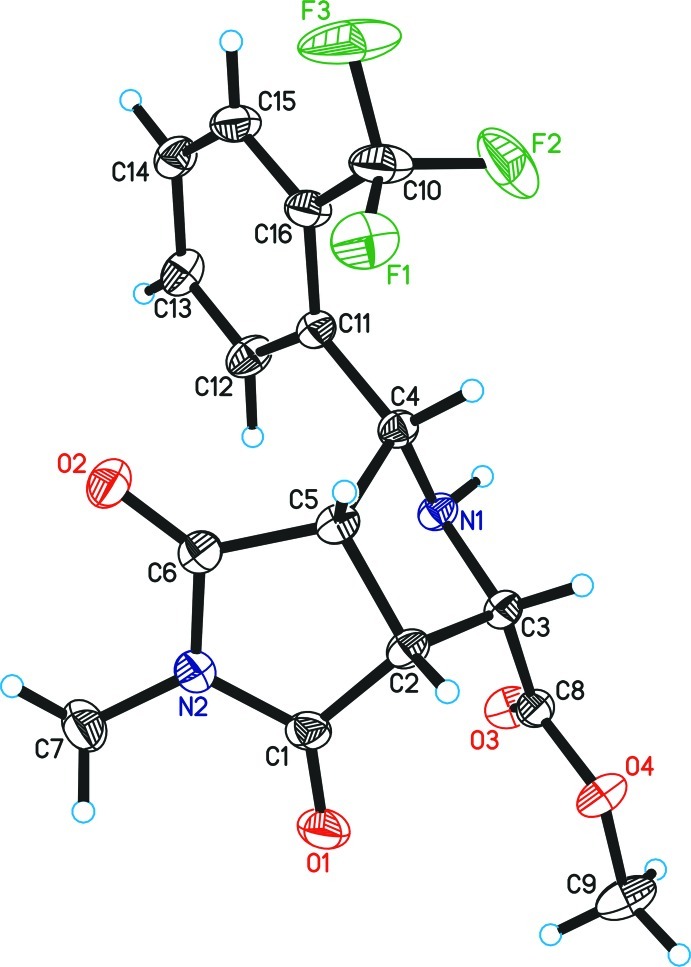
The molecular structure of the title compound, showing the numbering scheme adopted. Displacement ellipsoids are shown at the 50% probability level.

**Fig. 3.**
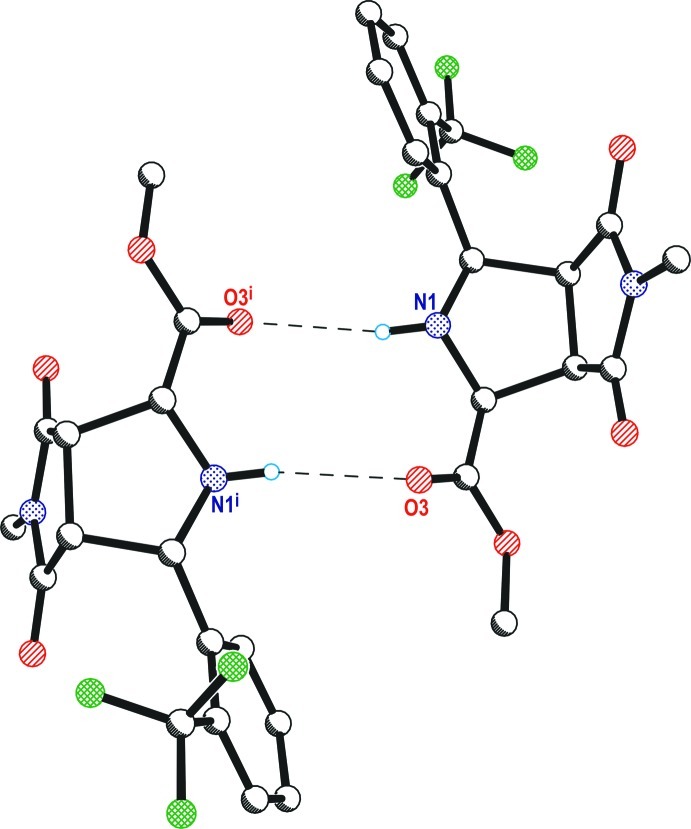
Hydrogen-bonded dimers in the structure of the title compound. H-bonds are shown as dashed lines. [Symmetry code: (i) 1 - x, 2 - y, 1 - z.]

## supplementary crystallographic information

### Comment

The core of the title compound is formed by two fused pyrrolidine cycles. It was effectively synthesized by the three-component approach developed by the authors (Fig. 1). Combination of molecular sieves and triethyamine represents an efficient reagent for performing three-component interaction of benzaldehyde, glycine ester and dipolarophile. The product X-ray structure determination indicates that cycloaddition step proceeds as *endo*-process (Fig. 2). Tetrasubstituted pyrrolidine cycle adopts envelope conformation with N1 atom occupying flap position. Amine atom N1 has trigonal pyramidal environment with the sum of valent angles equal to 329.0 °. Hydrogen atom H1 lies in axial position (relative to the mean plane of five-membered ring). As expected, dioxopyrrolidine cycle is planar within 0.0354 (7) Å.

In the crystal, the adjacent molecules are combined in centrosymmetric dimers by two weak N—H···O hydrogen bonds (Fig. 3). The same dimers were previously observed in the structure of (1*SR*,3*RS*,3a*SR*,6a*RS*-methyl 5-methyl-4,6-dioxo-3-phenyloctahydropyrrolo [3,4-*c*]pyrrole-1-carboxylate (Kudryavtsev & Zagulyaeva, 2008).

### Experimental

Triethylamine (0.340 ml, 2.41 mmol) was added to the stirred mixture of 2-(trifluoromethyl)benzaldehyde (200 mg, 1.15 mmol), *N*-methylmaleimide (130 mg, 1.15 mmol), glycine methyl ester hydrochloride (158 mg, 1.30 mmol) and 4 Å molecular sieves (200 mg) in toluene under argon atmosphere. Reaction mixture was stirred for 48 h. Volatiles were removed at reduced pressure. CH_2_Cl_2_ (50 ml) was added to the residue, resulted suspension was filtered through Celite, washed with saturated solution of NH_4_Cl (2 *x* 10 ml). Organic phase was dried over Na_2_SO_4_, concentrated and purified by column chromatography on silica gel 60 (particle size 0.040–0.063 mm) using CH_2_Cl_2_—MeOH (100:1) as eluent. Yield 168 mg (41%), colorless crystals, mp 183–185°C. ^1^H NMR (400 MHz, CDCl_3_): δ 2.61 (s, 3H, NCH_3_), 3.28 (t, 1H, H-3a, *J* 8.2), 3.40 (t, 1H, H-6a, *J* 7.3), 3.64 (s, 3H, OCH_3_), 3.95 (d, 1H, H-1, *J* 6.7), 4.59 (d, 1H, H-3, *J* 8.6), 7.20 (t, 1H, Ar, *J* 7.6), 7.30 (t, 1H, Ar, *J* 7.6), 7.61 (d, 1H, Ar, *J* 7.6). ^13^C NMR (100 MHz, CDCl_3_): δ 24.94, 47.31, 48.92, 52.26, 58.89, 61.04, 123.02, 125.89, 128.06, 128.23, 131.96, 135.74, 169.84, 174.30, 175.78. Found, %: C, 54.12; H, 4.27; N, 7.78. C_16_H_15_F_3_N_2_O_4_. Calculated, %: C, 53.94; H, 4.24; N, 7.86. The crystals were obtained by slow evaporation of the CDCl_3_ solution.

### Refinement

All hydrogen atoms were located in a difference Fourier map and refined with isotropic thermal parameters.

### Crystal data

**Table:**

C_16_H_15_F_3_N_2_O_4_	*F*(000) = 1472
*M**_r_* = 356.30	*D*_x_ = 1.506 Mg m^−^^3^
Orthorhombic, *P**b**c**a*	Mo *K*α radiation, λ = 0.71073 Å
Hall symbol: -P 2ac 2ab	Cell parameters from 7037 reflections
*a* = 11.6168 (4) Å	θ = 2.6–30.2°
*b* = 12.7385 (5) Å	µ = 0.13 mm^−^^1^
*c* = 21.2429 (8) Å	*T* = 150 K
*V* = 3143.5 (2) Å^3^	Block, colourless
*Z* = 8	0.35 × 0.30 × 0.25 mm

### Data collection

**Table:**

Bruker SMART APEXII diffractometer	4585 independent reflections
Radiation source: fine-focus sealed tube	3698 reflections with *I* > 2σ(*I*)
Graphite monochromator	*R*_int_ = 0.032
ω scans	θ_max_ = 30.0°, θ_min_ = 2.6°
Absorption correction: multi-scan (*SADABS*; Bruker, 2008)	*h* = −16→16
*T*_min_ = 0.955, *T*_max_ = 0.968	*k* = −17→16
28987 measured reflections	*l* = −29→29

### Refinement

**Table:**

Refinement on *F*^2^	Primary atom site location: structure-invariant direct methods
Least-squares matrix: full	Secondary atom site location: difference Fourier map
*R*[*F*^2^ > 2σ(*F*^2^)] = 0.041	Hydrogen site location: difference Fourier map
*wR*(*F*^2^) = 0.115	All H-atom parameters refined
*S* = 1.05	*w* = 1/[σ^2^(*F*_o_^2^) + (0.0599*P*)^2^ + 0.958*P*] where *P* = (*F*_o_^2^ + 2*F*_c_^2^)/3
4585 reflections	(Δ/σ)_max_ = 0.001
286 parameters	Δρ_max_ = 0.41 e Å^−^^3^
0 restraints	Δρ_min_ = −0.36 e Å^−^^3^

### Special details

**Table:**

Geometry. All e.s.d.'s (except the e.s.d. in the dihedral angle between two l.s. planes) are estimated using the full covariance matrix. The cell e.s.d.'s are taken into account individually in the estimation of e.s.d.'s in distances, angles and torsion angles; correlations between e.s.d.'s in cell parameters are only used when they are defined by crystal symmetry. An approximate (isotropic) treatment of cell e.s.d.'s is used for estimating e.s.d.'s involving l.s. planes.
Refinement. Refinement of *F*^2^ against ALL reflections. The weighted *R*-factor *wR* and goodness of fit *S* are based on *F*^2^, conventional *R*-factors *R* are based on *F*, with *F* set to zero for negative *F*^2^. The threshold expression of *F*^2^ > σ(*F*^2^) is used only for calculating *R*-factors(gt) *etc*. and is not relevant to the choice of reflections for refinement. *R*-factors based on *F*^2^ are statistically about twice as large as those based on *F*, and *R*- factors based on ALL data will be even larger.

### Fractional atomic coordinates and isotropic or equivalent isotropic displacement parameters (Å^2^)

**Table:**

	*x*	*y*	*z*	*U*_iso_*/*U*_eq_	
N1	0.37232 (8)	0.96741 (8)	0.58121 (4)	0.01832 (19)	
N2	0.43578 (9)	0.80090 (8)	0.70073 (5)	0.0223 (2)	
O1	0.59707 (8)	0.90401 (9)	0.70207 (5)	0.0323 (2)	
O2	0.25210 (8)	0.73656 (7)	0.69180 (4)	0.0293 (2)	
O3	0.60527 (8)	1.01872 (8)	0.56746 (4)	0.0261 (2)	
O4	0.59604 (7)	1.12962 (7)	0.64969 (4)	0.0265 (2)	
F1	0.02339 (7)	0.95536 (7)	0.66222 (4)	0.0360 (2)	
F2	0.01291 (10)	1.04045 (8)	0.57563 (5)	0.0519 (3)	
F3	−0.11317 (8)	0.92265 (11)	0.59782 (7)	0.0708 (4)	
C1	0.49344 (10)	0.89519 (10)	0.69792 (5)	0.0218 (2)	
C2	0.40611 (10)	0.98226 (9)	0.68818 (5)	0.0187 (2)	
C3	0.42693 (10)	1.03976 (9)	0.62520 (5)	0.0182 (2)	
C4	0.25821 (9)	0.94880 (9)	0.60901 (5)	0.0180 (2)	
C5	0.29053 (9)	0.92447 (9)	0.67894 (5)	0.0187 (2)	
C6	0.31822 (10)	0.80982 (9)	0.69077 (5)	0.0206 (2)	
C7	0.49500 (13)	0.70166 (11)	0.71063 (7)	0.0309 (3)	
C8	0.55262 (10)	1.05870 (9)	0.60979 (5)	0.0197 (2)	
C9	0.71865 (12)	1.14732 (14)	0.64520 (7)	0.0352 (3)	
C10	−0.00083 (11)	0.94442 (12)	0.60086 (7)	0.0342 (3)	
C11	0.19231 (9)	0.86470 (9)	0.57357 (5)	0.0182 (2)	
C12	0.25319 (10)	0.78516 (10)	0.54317 (6)	0.0238 (2)	
C13	0.19746 (11)	0.70890 (10)	0.50777 (6)	0.0246 (2)	
C14	0.07863 (11)	0.70963 (10)	0.50236 (6)	0.0241 (2)	
C15	0.01667 (11)	0.78587 (10)	0.53409 (6)	0.0265 (3)	
C16	0.07221 (10)	0.86314 (10)	0.56912 (6)	0.0224 (2)	
H4	0.2122 (13)	1.0153 (12)	0.6103 (7)	0.023 (4)*	
H14	0.0394 (13)	0.6570 (12)	0.4770 (7)	0.024 (4)*	
H5	0.2277 (12)	0.9465 (11)	0.7074 (7)	0.018 (3)*	
H1	0.3693 (12)	0.9935 (12)	0.5427 (7)	0.021 (4)*	
H3	0.3902 (13)	1.1102 (12)	0.6286 (7)	0.025 (4)*	
H15	−0.0638 (14)	0.7870 (13)	0.5322 (8)	0.034 (4)*	
H2	0.4071 (13)	1.0292 (13)	0.7231 (7)	0.028 (4)*	
H12	0.3371 (15)	0.7847 (13)	0.5480 (8)	0.034 (4)*	
H13	0.2416 (14)	0.6568 (13)	0.4868 (8)	0.032 (4)*	
H73	0.5522 (17)	0.7131 (15)	0.7416 (10)	0.051 (5)*	
H72	0.5263 (16)	0.6773 (15)	0.6724 (10)	0.048 (5)*	
H93	0.7376 (17)	1.2026 (16)	0.6754 (9)	0.053 (5)*	
H71	0.4386 (16)	0.6518 (15)	0.7264 (9)	0.043 (5)*	
H92	0.7571 (18)	1.0823 (17)	0.6561 (9)	0.052 (6)*	
H91	0.7395 (18)	1.1612 (16)	0.6019 (10)	0.057 (6)*	

### Atomic displacement parameters (Å^2^)

**Table:**

	*U*^11^	*U*^22^	*U*^33^	*U*^12^	*U*^13^	*U*^23^
N1	0.0192 (4)	0.0201 (5)	0.0156 (4)	−0.0034 (3)	−0.0001 (3)	−0.0012 (3)
N2	0.0226 (5)	0.0219 (5)	0.0223 (4)	−0.0002 (4)	−0.0016 (4)	0.0028 (4)
O1	0.0209 (4)	0.0408 (6)	0.0351 (5)	−0.0038 (4)	−0.0069 (4)	0.0116 (4)
O2	0.0306 (5)	0.0260 (5)	0.0313 (4)	−0.0092 (4)	−0.0024 (4)	0.0040 (4)
O3	0.0235 (4)	0.0328 (5)	0.0219 (4)	−0.0027 (4)	0.0024 (3)	−0.0044 (3)
O4	0.0226 (4)	0.0287 (5)	0.0282 (4)	−0.0085 (4)	0.0013 (3)	−0.0080 (4)
F1	0.0303 (4)	0.0427 (5)	0.0349 (4)	0.0047 (3)	0.0050 (3)	−0.0139 (4)
F2	0.0706 (7)	0.0358 (5)	0.0493 (6)	0.0292 (5)	−0.0191 (5)	−0.0091 (4)
F3	0.0184 (4)	0.0833 (8)	0.1107 (10)	0.0122 (5)	−0.0119 (5)	−0.0622 (8)
C1	0.0222 (5)	0.0272 (6)	0.0162 (5)	−0.0026 (4)	−0.0030 (4)	0.0019 (4)
C2	0.0199 (5)	0.0206 (5)	0.0157 (4)	−0.0035 (4)	0.0002 (4)	−0.0033 (4)
C3	0.0191 (5)	0.0176 (5)	0.0178 (4)	−0.0020 (4)	−0.0002 (4)	−0.0017 (4)
C4	0.0169 (5)	0.0184 (5)	0.0185 (5)	−0.0004 (4)	0.0001 (4)	−0.0024 (4)
C5	0.0172 (5)	0.0215 (5)	0.0174 (4)	−0.0020 (4)	0.0016 (4)	−0.0021 (4)
C6	0.0223 (5)	0.0235 (6)	0.0161 (5)	−0.0027 (4)	0.0010 (4)	0.0000 (4)
C7	0.0309 (7)	0.0268 (7)	0.0352 (7)	0.0057 (5)	−0.0003 (5)	0.0065 (5)
C8	0.0207 (5)	0.0195 (5)	0.0189 (5)	−0.0022 (4)	−0.0014 (4)	0.0009 (4)
C9	0.0232 (6)	0.0457 (9)	0.0367 (7)	−0.0127 (6)	0.0005 (5)	−0.0083 (6)
C10	0.0202 (6)	0.0376 (8)	0.0448 (8)	0.0082 (5)	−0.0101 (5)	−0.0172 (6)
C11	0.0182 (5)	0.0191 (5)	0.0174 (5)	−0.0019 (4)	−0.0003 (4)	−0.0009 (4)
C12	0.0186 (5)	0.0255 (6)	0.0274 (5)	−0.0036 (4)	0.0050 (4)	−0.0062 (4)
C13	0.0254 (6)	0.0226 (6)	0.0257 (5)	−0.0034 (5)	0.0065 (4)	−0.0061 (4)
C14	0.0262 (6)	0.0228 (6)	0.0231 (5)	−0.0045 (5)	−0.0024 (4)	−0.0039 (4)
C15	0.0198 (5)	0.0290 (6)	0.0307 (6)	0.0000 (5)	−0.0065 (5)	−0.0059 (5)
C16	0.0185 (5)	0.0234 (6)	0.0254 (5)	0.0036 (4)	−0.0044 (4)	−0.0048 (4)

### Geometric parameters (Å, º)

**Table:**

N1—C3	1.4578 (14)	C4—C5	1.5633 (15)
N1—C4	1.4704 (14)	C4—H4	1.002 (15)
N1—H1	0.884 (15)	C5—C6	1.5164 (17)
N2—C1	1.3766 (16)	C5—H5	0.988 (14)
N2—C6	1.3866 (15)	C7—H73	0.95 (2)
N2—C7	1.4545 (16)	C7—H72	0.94 (2)
O1—C1	1.2124 (14)	C7—H71	0.972 (19)
O2—C6	1.2089 (15)	C9—H93	0.98 (2)
O3—C8	1.2008 (14)	C9—H92	0.97 (2)
O4—C8	1.3375 (14)	C9—H91	0.97 (2)
O4—C9	1.4451 (15)	C10—C16	1.4990 (17)
F1—C10	1.3406 (17)	C11—C12	1.3942 (16)
F2—C10	1.3452 (19)	C11—C16	1.3984 (15)
F3—C10	1.3357 (16)	C12—C13	1.3886 (16)
C1—C2	1.5173 (17)	C12—H12	0.980 (17)
C2—C5	1.5437 (15)	C13—C14	1.3852 (17)
C2—C3	1.5444 (15)	C13—H13	0.950 (17)
C2—H2	0.953 (16)	C14—C15	1.3842 (18)
C3—C8	1.5157 (16)	C14—H14	0.973 (15)
C3—H3	0.997 (16)	C15—C16	1.3923 (17)
C4—C11	1.5168 (15)	C15—H15	0.936 (17)
			
C3—N1—C4	103.70 (8)	N2—C7—H72	110.1 (12)
C3—N1—H1	112.0 (10)	H73—C7—H72	112.3 (16)
C4—N1—H1	113.3 (9)	N2—C7—H71	107.4 (11)
C1—N2—C6	113.64 (10)	H73—C7—H71	109.5 (16)
C1—N2—C7	122.31 (11)	H72—C7—H71	110.0 (16)
C6—N2—C7	124.00 (11)	O3—C8—O4	124.67 (11)
C8—O4—C9	115.80 (10)	O3—C8—C3	125.80 (10)
O1—C1—N2	124.12 (12)	O4—C8—C3	109.51 (9)
O1—C1—C2	127.31 (11)	O4—C9—H93	106.9 (12)
N2—C1—C2	108.57 (10)	O4—C9—H92	107.7 (12)
C1—C2—C5	104.50 (9)	H93—C9—H92	110.8 (17)
C1—C2—C3	111.11 (9)	O4—C9—H91	109.7 (12)
C5—C2—C3	104.61 (9)	H93—C9—H91	115.8 (17)
C1—C2—H2	110.2 (10)	H92—C9—H91	105.6 (17)
C5—C2—H2	114.1 (10)	F3—C10—F1	105.89 (13)
C3—C2—H2	112.0 (10)	F3—C10—F2	106.58 (12)
N1—C3—C8	112.42 (9)	F1—C10—F2	105.55 (11)
N1—C3—C2	100.80 (9)	F3—C10—C16	112.82 (11)
C8—C3—C2	114.43 (9)	F1—C10—C16	112.98 (11)
N1—C3—H3	115.4 (9)	F2—C10—C16	112.44 (13)
C8—C3—H3	106.6 (9)	C12—C11—C16	117.67 (10)
C2—C3—H3	107.3 (9)	C12—C11—C4	119.15 (10)
N1—C4—C11	111.69 (9)	C16—C11—C4	123.17 (10)
N1—C4—C5	101.36 (8)	C13—C12—C11	121.52 (11)
C11—C4—C5	116.93 (9)	C13—C12—H12	121.0 (10)
N1—C4—H4	110.8 (9)	C11—C12—H12	117.4 (10)
C11—C4—H4	109.9 (9)	C14—C13—C12	120.32 (11)
C5—C4—H4	105.7 (8)	C14—C13—H13	120.2 (10)
C6—C5—C2	104.70 (9)	C12—C13—H13	119.4 (10)
C6—C5—C4	113.54 (9)	C15—C14—C13	118.85 (11)
C2—C5—C4	103.61 (9)	C15—C14—H14	120.6 (9)
C6—C5—H5	109.2 (8)	C13—C14—H14	120.5 (9)
C2—C5—H5	115.4 (8)	C14—C15—C16	121.01 (11)
C4—C5—H5	110.4 (8)	C14—C15—H15	120.6 (10)
O2—C6—N2	124.03 (11)	C16—C15—H15	118.4 (10)
O2—C6—C5	127.72 (11)	C15—C16—C11	120.57 (11)
N2—C6—C5	108.25 (9)	C15—C16—C10	117.79 (11)
N2—C7—H73	107.4 (12)	C11—C16—C10	121.63 (11)

### Hydrogen-bond geometry (Å, º)

**Table:**

*D*—H···*A*	*D*—H	H···*A*	*D*···*A*	*D*—H···*A*
N1—H1···O3^i^	0.884 (15)	2.363 (15)	3.1738 (13)	152.5 (13)

Symmetry code: (i) −*x*+1, −*y*+2, −*z*+1.
